# Effect of serum MMP-7 on the diagnostic accuracy of biliary atresia: systematic review and meta-analysis

**DOI:** 10.3389/fphar.2025.1581053

**Published:** 2025-06-26

**Authors:** Haojun Wang, Hui Liu, Wanfu Li, Ainiwaer Keranmu, Peng Liang, Yaqi Wang, Tao Zhang, Yeliaman Jiayilawu

**Affiliations:** Department of Pediatric General Surgery, The First Affiliated Hospital of Xinjiang Medical University, Urumqi, Xinjiang, China

**Keywords:** biliary atresia, MMP-7, diagnostic, cirrhosis, meta-analysis

## Abstract

**Background:**

Biliary atresia (BA) is an obstructive fibrotic disorder that typically progresses to cirrhosis and, ultimately, death in children. Current gold standard diagnostics for BA include intraoperative cholangiography and liver biopsy, both invasive procedures with significant complication risks. Matrix metalloproteinase-7 (MMP-7) has emerged as a highly accurate noninvasive diagnostic biomarker for BA. This study therefore aimed to evaluate the diagnostic utility of serum MMP-7 for BA detection.

**Methods:**

We performed a comprehensive search of English-language databases (PubMed, Web of Science, ScienceDirect) with literature from the inception of these databases through 30 November 2024. Study quality was assessed using the QUADAS-2 tool. Sensitivity, specificity, positive/negative likelihood ratios (PLR/NLR), and area under the receiver operating characteristic curve (AUC-ROC) were calculated using Meta-DiSc 1.4 and STATA 18.0.

**Results:**

This systematic review and meta-analysis included 13 articles (17 studies) comprising 2,836 serum samples from pediatric subjects. Binary classification model analysis showed pooled sensitivity of 0.93 (95% CI: 0.92–0.94) and specificity of 0.85 (95% CI: 0.83–0.87) for MMP-7. Positive likelihood ratio (PLR) was 7.68 (95%CI: 5.04–11.72), the negative likelihood ratio (NLR) was 0.08 (95%CI: 0.05–0.14), and the diagnosis odds ratio (DOR) was 104.34 (95%CI: 55.97–194.51). AUC was 0.9628. Meta-regression analysis identified publication year as a significant heterogeneity source (p = 0.007). Sensitivity analysis confirmed the robust diagnostic stability of MMP-7 for BA. Significant heterogeneity was observed across studies (I^2^ = 78.6%). Subgroup analysis by publication year showed that heterogeneity primarily originated from studies published in or after 2023.

**Conclusion:**

Serum MMP-7 represents a convenient, accurate, and reliable noninvasive biomarker for enhancing BA diagnostic efficiency. However, due to significant heterogeneity, further validation via large-scale, multicenter studies with standardized protocols is needed.

**Systematic Review Registration:**

https://www.crd.york.ac.uk/PROSPERO/recorddashboard, identifier CRD42024623643.

## Introduction

Biliary atresia (BA) is a severe obstructive fibroinflammatory disorder affecting extrahepatic and intrahepatic bile ducts (Fawaz et al., 2017). Without timely intervention, BA typically progresses to cirrhosis and ultimately fatal outcomes in children (Kerola et al., 2019). Therefore, early and accurate diagnosis is paramount in improving this condition’s prognosis.

Intraoperative cholangiography and liver biopsy are the gold standards for BA diagnosis and hepatic fibrosis evaluation. However, these invasive procedures carry significant complication risks, necessitating their use despite patient and parental burden (Lai and Afdhal, 2019; Singh et al., 2022). This underscores the urgent need for non-invasive diagnostic biomarkers to enhance early detection and management.

Emerging evidence identifies matrix metalloproteinases (MMPs) as critical enzymes in extracellular matrix degradation and remodelling, with roles in tissue remodelling, fibrosis, and pathological processes (Yao et al., 2023). Matrix metalloproteinase-7 (MMP-7) has drawn significant attention for its role in liver pathology. Studies demonstrate MMP-7 overexpression in liver biopsies from BA children, with clear differentiation from non-liver-disease control groups (Hua et al., 2005). A large-scale quantitative serum proteomics study further revealed marked MMP-7 elevation in BA patients (Lertudomphonwanit et al., 2017). Notably, MMP-7 expression levels correlate significantly with BA-associated liver fibrosis, further underscoring its biomarker potential (Lertudomphonwanit et al., 2017; Yang et al., 2018; Chi et al., 2022; Wu et al., 2019).

While MMP-7 demonstrates diagnostic promise, most studies are limited by single-center designs and small sample sizes, which compromise generalizability and clinical relevance. Current MMP-7 applications focus on postnatal differential diagnosis in jaundiced or cholestatic infants, though standardized timing protocols for testing remain unestablished. Notably, only one study (Jiang et al., 2024) conducted stratified analysis in 0–30-day-old infants, defining an optimal cutoff value of 28.1 ng/mL (sensitivity: 86.4%, specificity: 95.5%). Prenatal applications of MMP-7, such as cord blood or amniotic fluid analysis, are unexplored in current literature. However, given the potential fetal origins of BA pathogenesis, future research should investigate MMP-7 integration into prenatal screening algorithms. Due to pronounced age-related heterogeneity in BA presentations (e.g., variable hepatic fibrosis severity in neonates vs infants), stratified analyses of MMP-7 diagnostic thresholds and performance across age brackets (0–2 weeks, 2–4 weeks, 1–3 months, >3 months) are strongly recommended. Such stratification would facilitate the development of more accurate clinical decision benchmarks.

This study aims to systematically evaluate the diagnostic accuracy of serum MMP-7 for BA and assess its clinical applicability through meta-analytic integration of large-sample study data. This research provides evidence to establish MMP-7 as a reliable noninvasive biomarker for early BA diagnosis and prognostic evaluation in pediatric populations.

## Methods

This study has been registered in PROSPERO, ID: CRD42024623643.

### Study inclusion

We searched both databases (PubMed, ScienceDirect, Web of Science) for studies on the accuracy of MMP-7 in the diagnosis of BA. The search keywords included “Biliary atresia”, “Matrix metalloproteinase-7″, “MMP-7”, and “diagnosis”. The search covered publications from the inception of each database up to 30 November 2024. Only studies published in English were included.

### Inclusion and exclusion criteria

#### Inclusion criteria

Original studies evaluating the accuracy of serum MMP-7 in diagnosing BA.

Studies that provide sufficient information to construct a 2 × 2 contingency table. Studies using intraoperative cholangiography or liver biopsy are the gold standard for diagnosing BA—publications from the databases’ inception until 30 November 2024.

#### Exclusion criteria

Animal studies, communications, dissertations, books, case reports, conference abstracts, journal reviews, expert opinions, studies with significant data errors, or screening-type studies.

Duplicate data or secondary publications were included—studies where 2 × 2 contingency table data could not be extracted.

### Selection process

Three researchers (HJW, HL and WFL) independently reviewed titles and abstracts of the records and discussed inconsistencies until consensus was obtained. Then, in pairs, the researchers independently screened the titles and abstracts of all articles retrieved. In case of disagreement, the discussion reached a consensus on which articles, the full text should be screened. Two researchers (HJW and HL) independently screened full-text articles for inclusion. Again, in case of disagreement, consensus was reached on inclusion or exclusion by discussion, and if necessary, the third researcher (WJL) was consulted.

### Data extraction and management

We included data only from children with jaundice, cholestasis, or neonatal hepatitis, excluding data from regular (healthy) populations. Studies using consistent detection methods were chosen. Data extraction was carried out independently by two researchers, and discrepancies were resolved through consultation with a third researcher. The extracted data included the following: study title, year of publication, country of origin, study period, types of research methods, study centre type, diagnostic gold standard, other diagnostic methods (excluding liver biopsy and laparoscopic cholangiography), inclusion and exclusion criteria, and detailed information on MMP-7, including total sample size, sample type, testing time, serum storage temperature and diagnostic threshold. The following diagnostic performance indicators were extracted: sensitivity, specificity, true positive (TP), false positive (FP), false negative (FN), and true negative (TN) values and the area under the receiver operating characteristic curve (AUC-ROC). If additional details were required for any study, we contacted the authors directly; if the authors did not respond or provide further information, that study was excluded to ensure the completeness and consistency of the data.

### Methodological quality assessment

The Quality Assessment of Diagnostic Accuracy Studies (QUADAS-2) tool was employed to evaluate the methodological quality of the studies, with a focus on bias risk and clinical applicability (Lee et al., 2022). The QUADAS-2 tool assesses four domains: patient selection, index test, reference standard, and flow and timing.

### Statistical analysis

All the data were entered into Microsoft 365 Excel (version 2,402) and independently verified by two researchers to ensure accuracy. Data analysis and visualisation were conducted via Meta-DiSc 1.4 (Zamora et al., 2006) and STATA 18.0 (Nyaga and Arbyn, 2024). For each primary study and its associated metrics, a 2 × 2 contingency table was constructed to calculate sensitivity, specificity, diagnosis odds ratio and their corresponding 95% confidence intervals. A diagnostic meta-analysis was performed via a bivariate model. Additionally, summary receiver operating characteristic (ROC) curves were generated for MMP-7, with the summary operating points of sensitivity and specificity clearly labelled.

### Overall diagnostic value of MMP-7 for BA

The heterogeneity assessment was initially performed by generating Receiver Operating Characteristic (ROC) curves based on the included studies and calculating Spearman’s correlation coefficient alongside the corresponding p-value (Author Anonymous, 2018). The heterogeneity across studies was further quantified using the I^2^ statistic, computed via Stata 18.0 software. When the I^2^ value exceeded 50%, heterogeneity was considered substantial, and a random-effects model was employed for analysis. Conversely, heterogeneity was deemed low if the I^2^ value was 50% or less, and a fixed-effects model was applied. In instances of significant heterogeneity (I^2^ > 50%), further investigation into potential sources of variation was undertaken through meta-regression analysis, sensitivity analysis and subgroup analysis (Händel et al., 2023). Publication bias was assessed using Deek’s funnel-plot asymmetry test, where a p-value ≤0.05 indicated the presence of significant publication bias, while a p-value >0.05 suggested its absence (Sterne et al., 2011). For cases where publication bias was detected, additional analyses were performed using Egger’s and Begg’s tests to provide more detailed insights. Finally, Fagan nomograms were constructed to estimate and visually depict the pre-test and post-test probabilities of MMP-7 detection, offering valuable diagnostic insights (Pötsch et al., 2022).

## Results

### Literature selection and quality assessment

A comprehensive search of English-language databases identified 152 articles. After rigorous screening and exclusion of studies not meeting predefined inclusion criteria, 13 articles were included in this systematic review. These articles originated from various countries, with contributions as follows: 6 from China (Yang et al., 2018; Chi et al., 2022; Wu et al., 2019; Xu et al., 2024; Jiang et al., 2024; Jiang et al., 2019), 2 from the United States (7,21), 1 from Japan (Sakaguchi et al., 2022), 1 from the United Kingdom (Aldeiri et al., 2023), 2 from Iran (Rohani et al., 2022; Karbasian et al., 2023), and 1 from India (Singh et al., 2022). Notably, two articles (Jiang (Jiang et al., 2019) and Pandurangi (Sakaguchi et al., 2022)) each contributed three studies, bringing the total to 17 studies.

**FIGURE 1:**
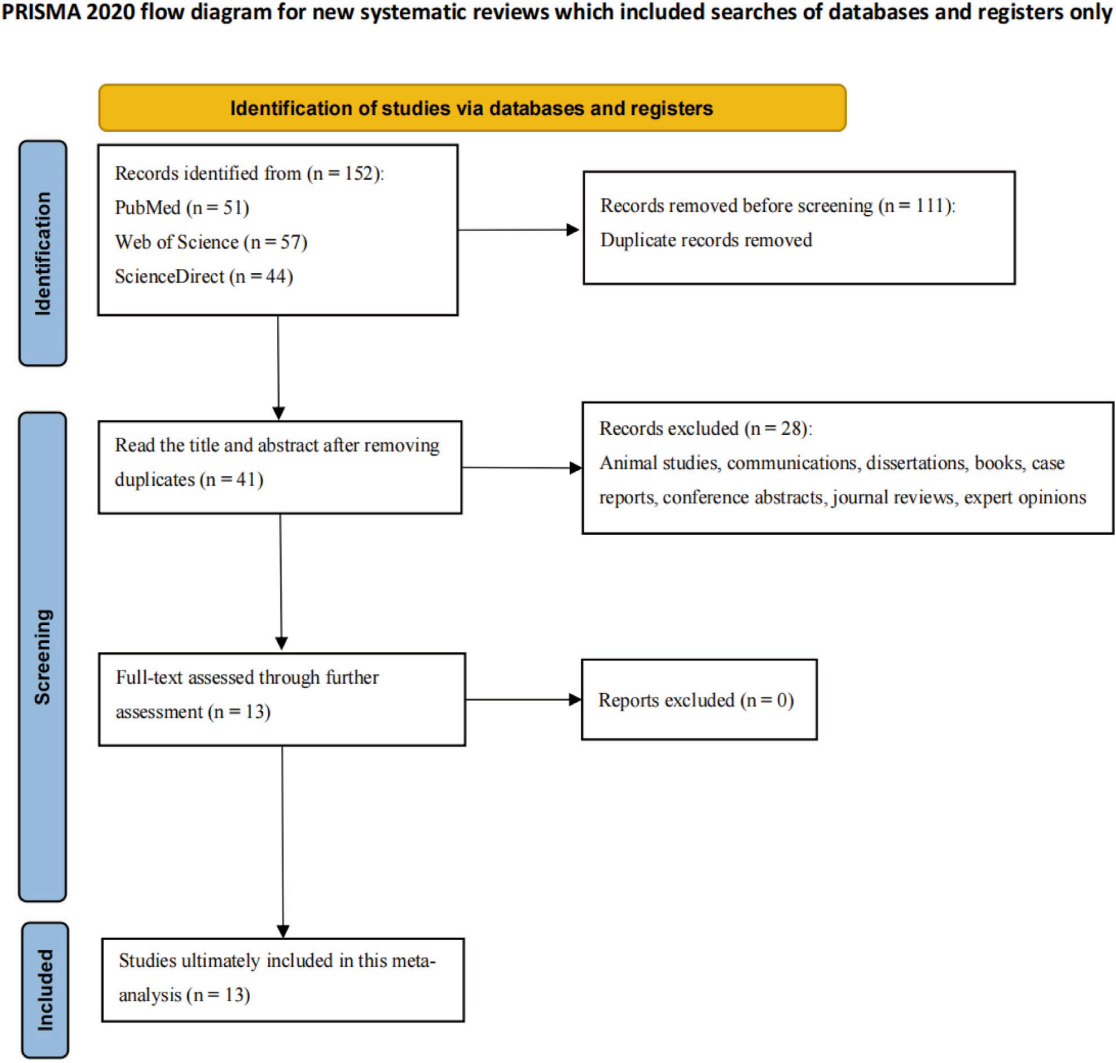
Study flow diagram.

Reported BA prevalence across included studies varied significantly (29.5%–64.9%), with a mean of 54.2%. Detailed study characteristics (design, demographics, diagnostic approaches) are listed in Supplementary Table S1 (Supplementary Material). Figure 1 depicts the literature screening and selection process, illustrating the systematic approach to ensure inclusion of high-quality, relevant studies.

The 17 included studies were published between 2017 and 2024, spanning six countries: China, United States, Japan, United Kingdom, India, and Iran. Study populations included infants diagnosed with postnatal jaundice or cholestasis. All studies used gold standard methods (intraoperative cholangiography/liver biopsy) for BA diagnosis, including 1,537 B A children (three studies by Pandurangi et al. (Pandurangi et al., 2024) shared the same sample group). Individual study sample sizes ranged from 13 to 395 children.

**FIGURE 2:**
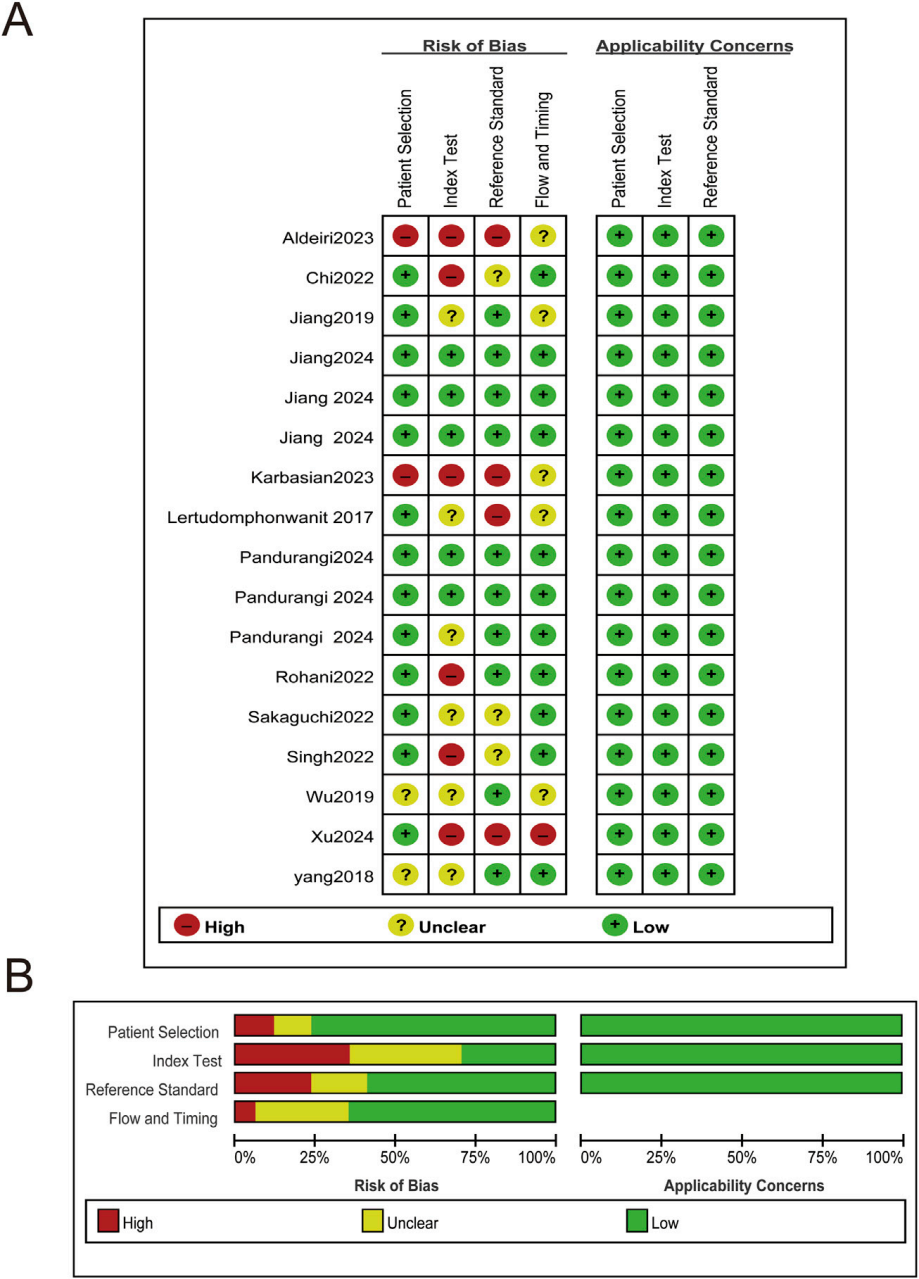
Risk of bias and applicability concerns plot. **(A)** Risk of bias and applicability concerns summary. **(B)** Risk of bias and applicability concerns graph.

Risk of bias for each study was assessed using the QUADAS-2 tool. Specific risk of bias assessments are shown in Figure 2A, with overall assessments in Figure 2B.

### Overall diagnostic value of MMP-7 for BA

**FIGURE 3:**
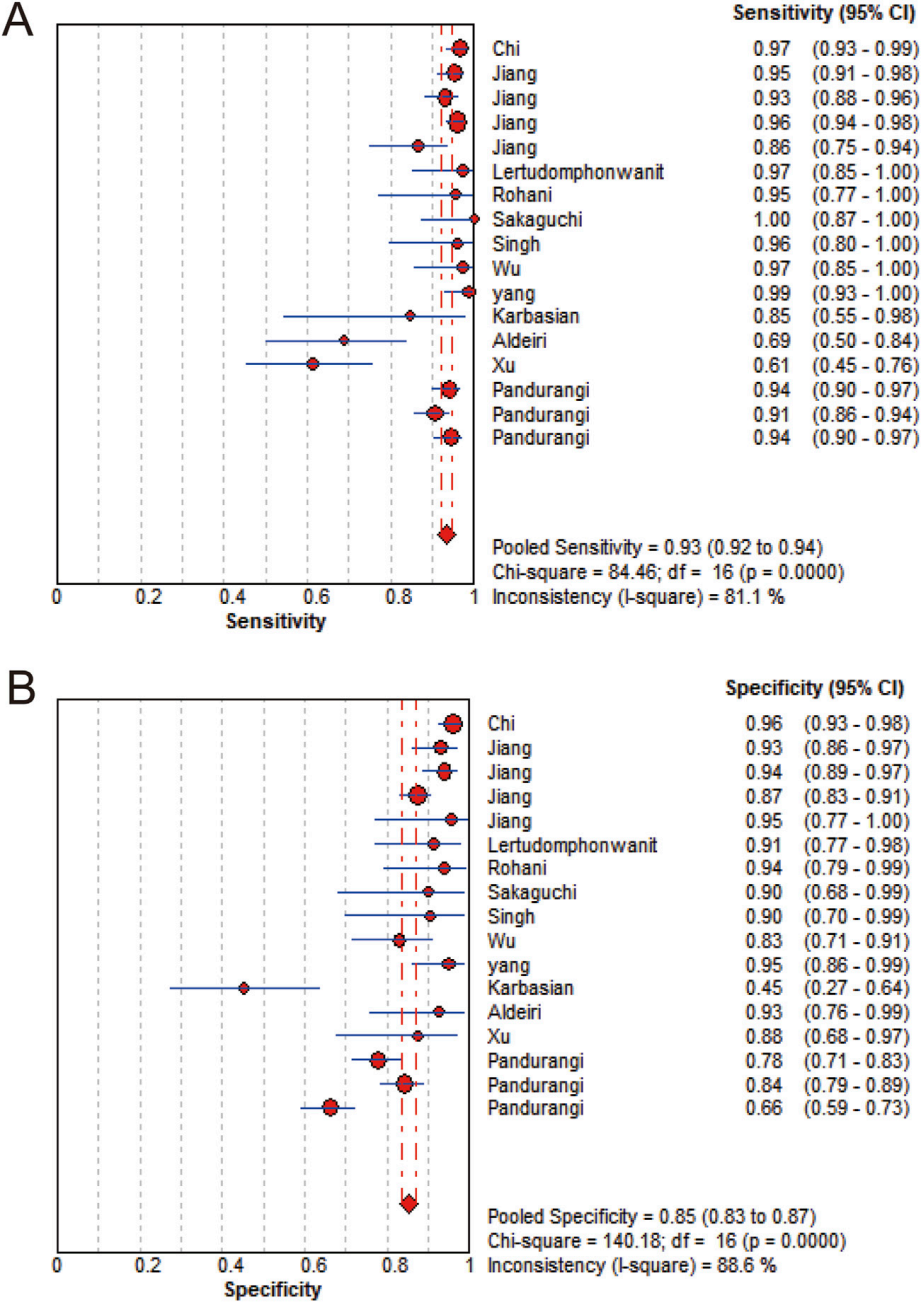
Forest plot. **(A)** Sensitivity forest. **(B)** Specificity forest.

**FIGURE 4:**
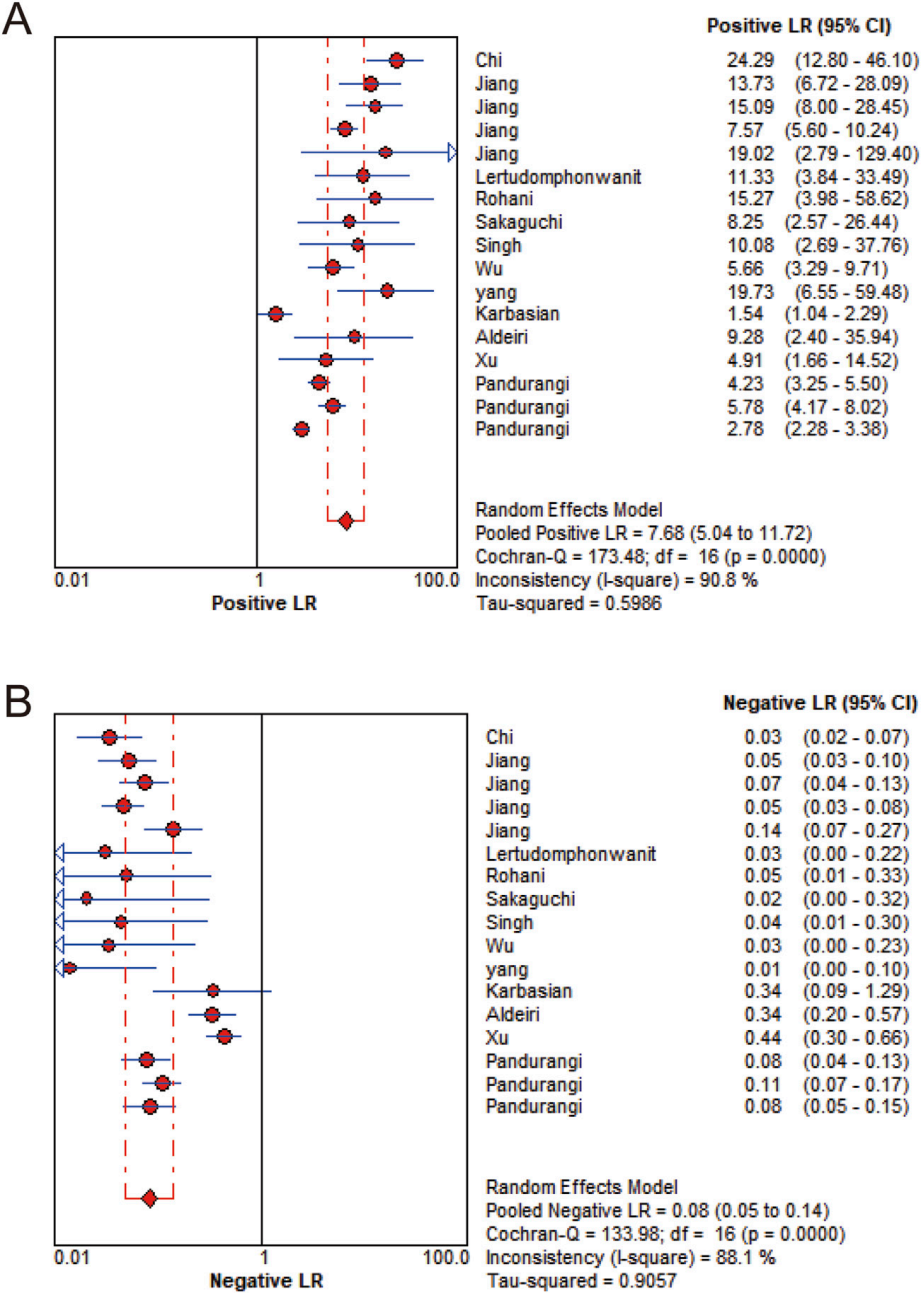
Likelihood ratio forest plot. **(A)** Positive likelihood ratio. **(B)** Negative likelihood ratio.

**FIGURE 5:**
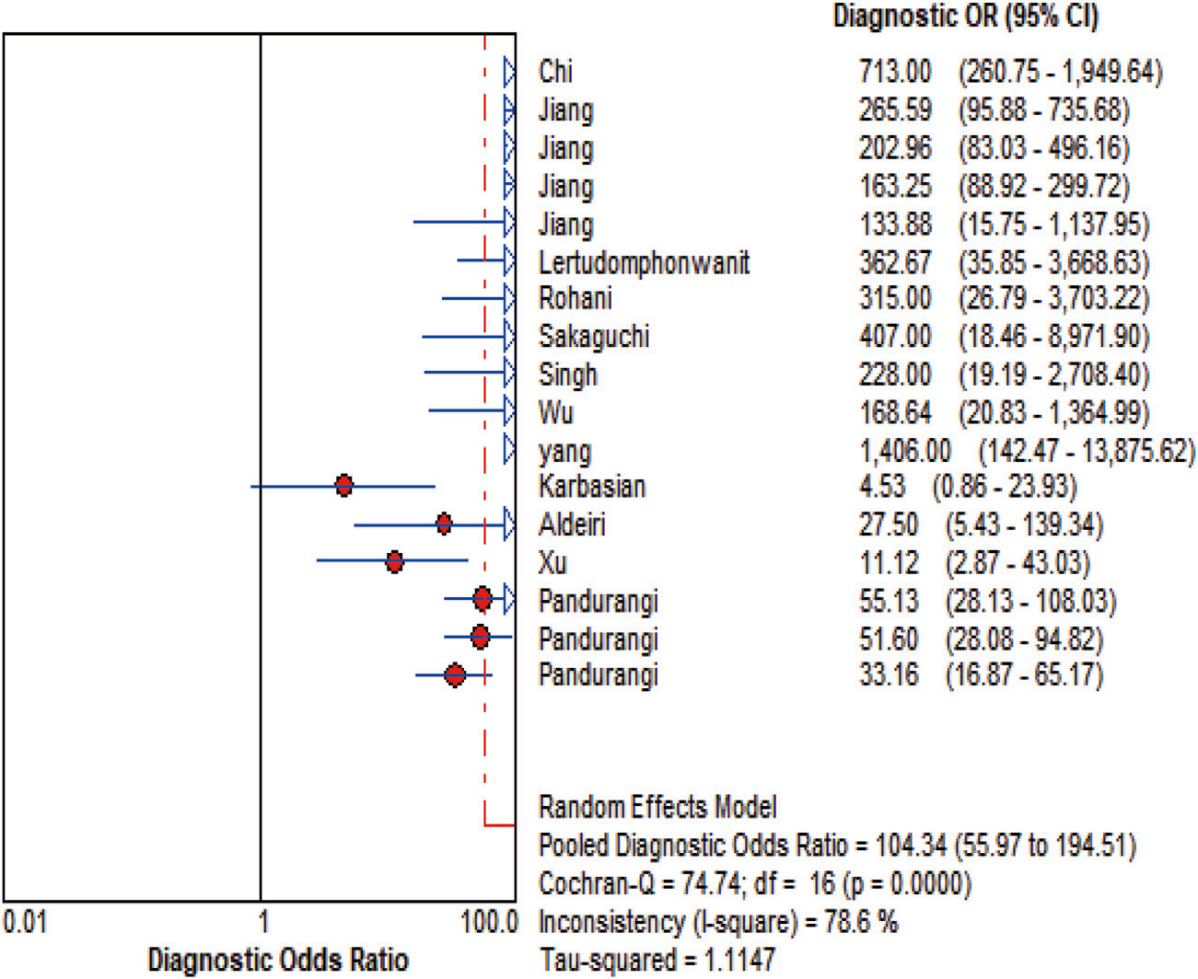
DOR Forest plot. The figure illustrates that the diagnostic odds ratio is 104.34 (55.97-194.51).

**FIGURE 6:**
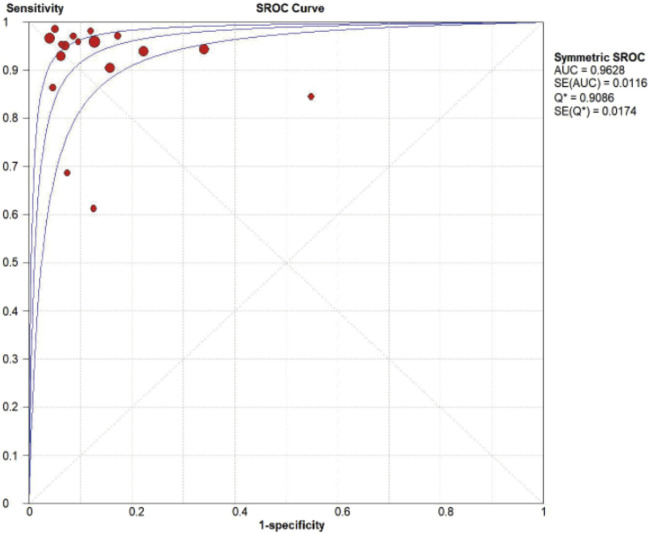
Summary receiver operating characteristic curve diagnosis of BA.

MMP-7 demonstrated excellent diagnostic sensitivity and specificity for BA, though substantial inter-study heterogeneity was observed. Pooled sensitivity was 0.93 (95% CI: 0.92–0.94), specificity 0.85 (95% CI: 0.83–0.87) (Figures 3A,B); PLR was 7.68 (95% CI: 5.04–11.72), NLR 0.08 (95% CI: 0.05–0.14) (Figures 4A,B); DOR was 104.34 (95% CI: 55.97–194.51) (Figure 5). However, significant heterogeneity was observed in DOR (I^2^ = 78.6%, Figure 5). Sensitivity and specificity showed high heterogeneity (I^2^ = 81.1% and 88.6%, respectively, Figures 3A,B). Spearman’s correlation analysis showed a non-significant threshold effect (correlation coefficient = −0.199, p = 0.445). The summary ROC curve (SROC) showed an AUC of 0.96, confirming MMP-7’s high diagnostic accuracy for BA (Figure 6).

### Investigation of heterogeneity and publication bias

Diagnostic meta-analysis heterogeneity may stem from threshold effects, covariate influences, or publication bias. Significant heterogeneity was observed among included studies, though no threshold effect was evident (correlation coefficient = −0.199, P = 0.445). Meta-regression analysis examined covariates including publication country, year, total sample size, BA sample size, testing method, and MMP-7 cut-off value. Publication year was identified as a significant heterogeneity source (P = 0.007).

**FIGURE 7:**
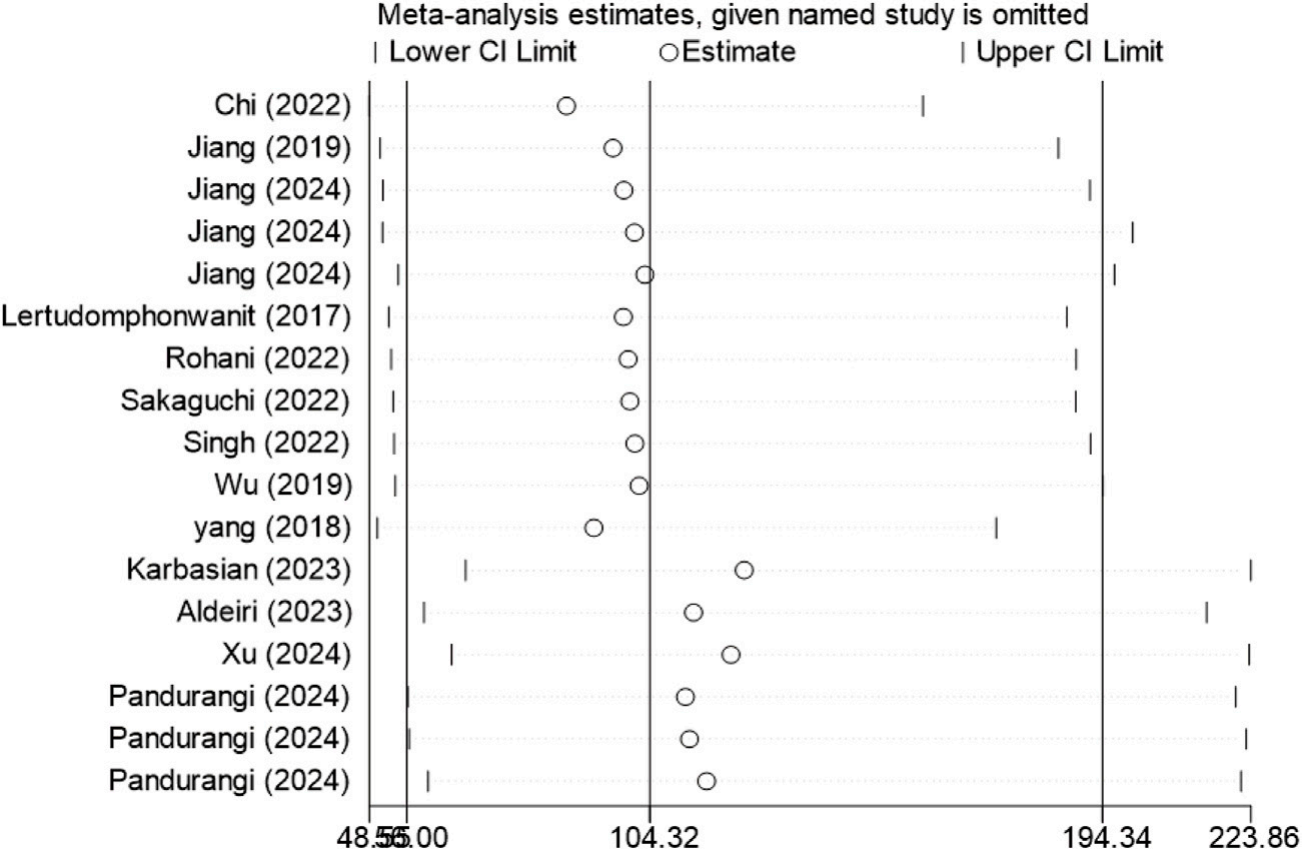
Sensitivity analysis of included studies. After removing any one study, the overall stability of the remaining studies is essentially unchanged.

Sensitivity analysis was performed using Stata 18.0 to assess robustness. This entailed sequential removal of individual studies, followed by pooled analysis of the remaining data. Results showed consistent overlap of diagnostic parameter confidence intervals with original data, indicating stability and reliability of included studies (Figure 7).

**FIGURE 8:**
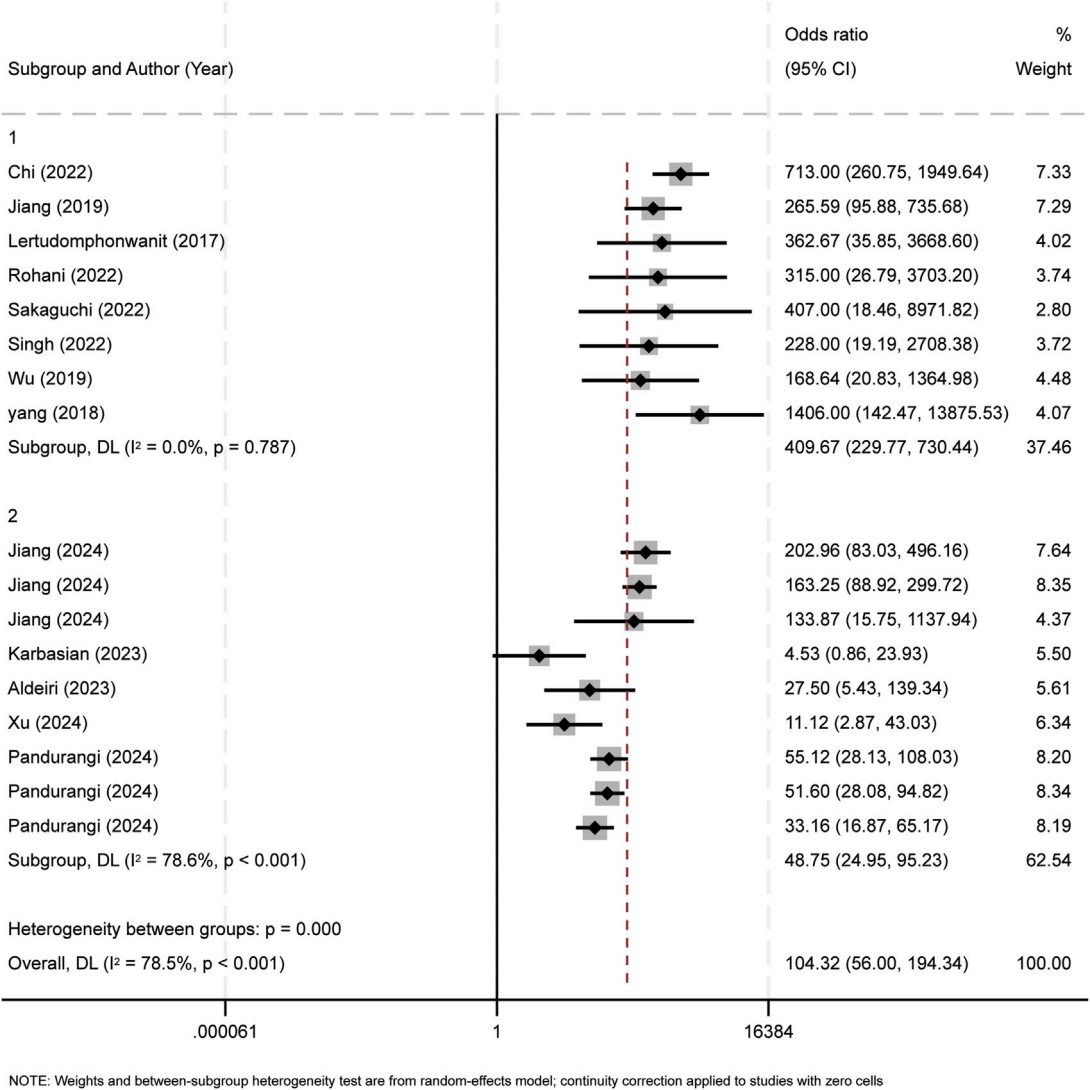
Subgroup analysis of publication year. The observed heterogeneity among the subgroups can be attributed to the findings from the studies conducted in 2023 and 2024.

**FIGURE 9:**
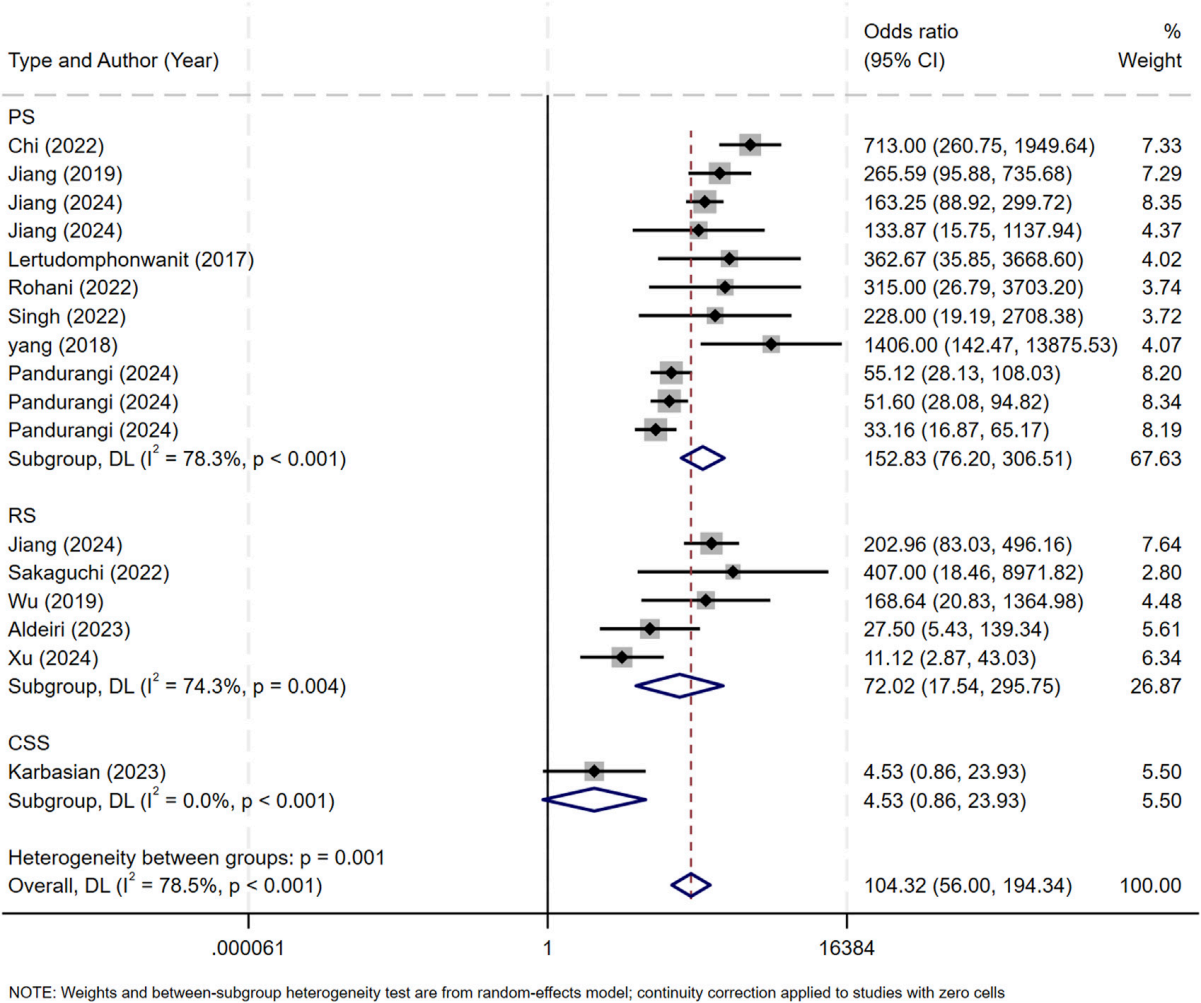
Subgroup analysis of research types. RS, retrospective study; PS, prospective study, CSS, cross - sectional study.

Subgroup analyses were performed according to two criteria: the median publication year (2023) and research method types, including retrospective study (RS), prospective study (PS), and cross - sectional study (CSS). Studies published before 2023 were assigned to Group 1, and those from 2023 onward to Group 2 (Figure 8). Subgroup analysis by research method types are shown in Figure 9.

**FIGURE 10:**
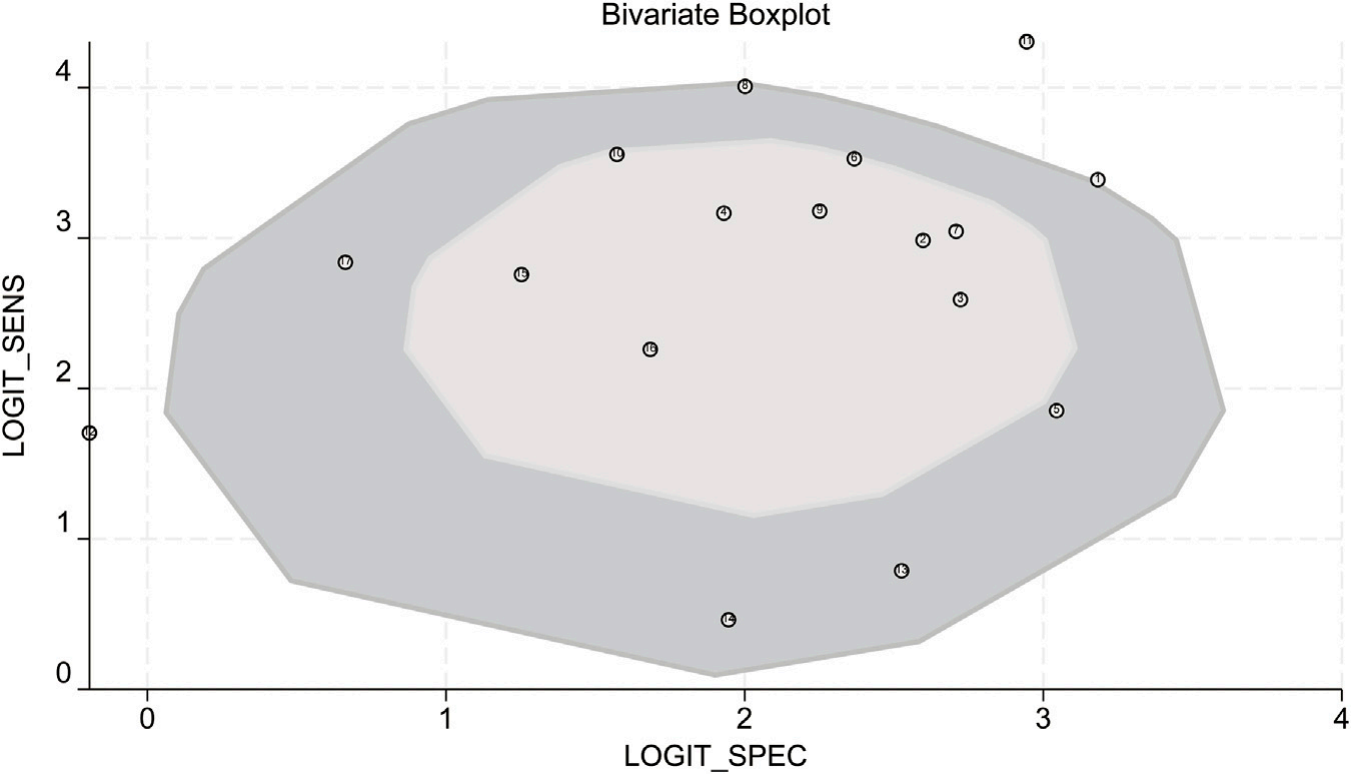
Bivariate boxplot. LOGIT_SENS: log-odds transformation of sensitivity; LOGIT_SPEC: log-odds transformation of specificity.

Bivariate box plot analysis identified three studies as potential outliers based on sensitivity and specificity distributions (Figure 10) (Yang et al., 2018; Chi et al., 2022; Karbasian et al., 2023).

**FIGURE 11:**
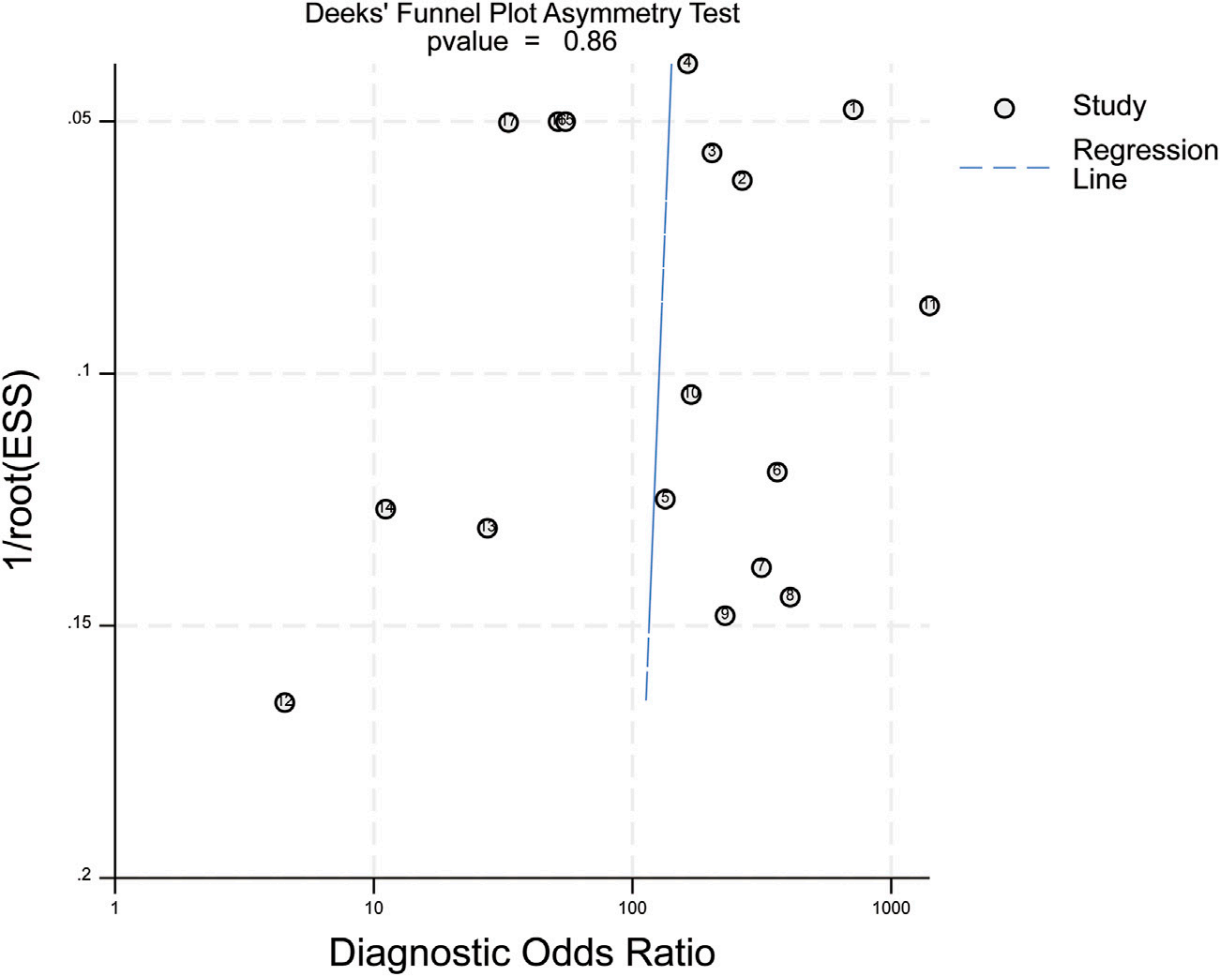
Deek’s funnel plot. ESS (Effective Sample Size): adequate sample size; P = 0.86 in the above figure indicates no publication bias in the included studies.

Deek’s funnel-plot asymmetry test showed no significant publication bias (P = 0.86, Figure 11).

**FIGURE 12:**
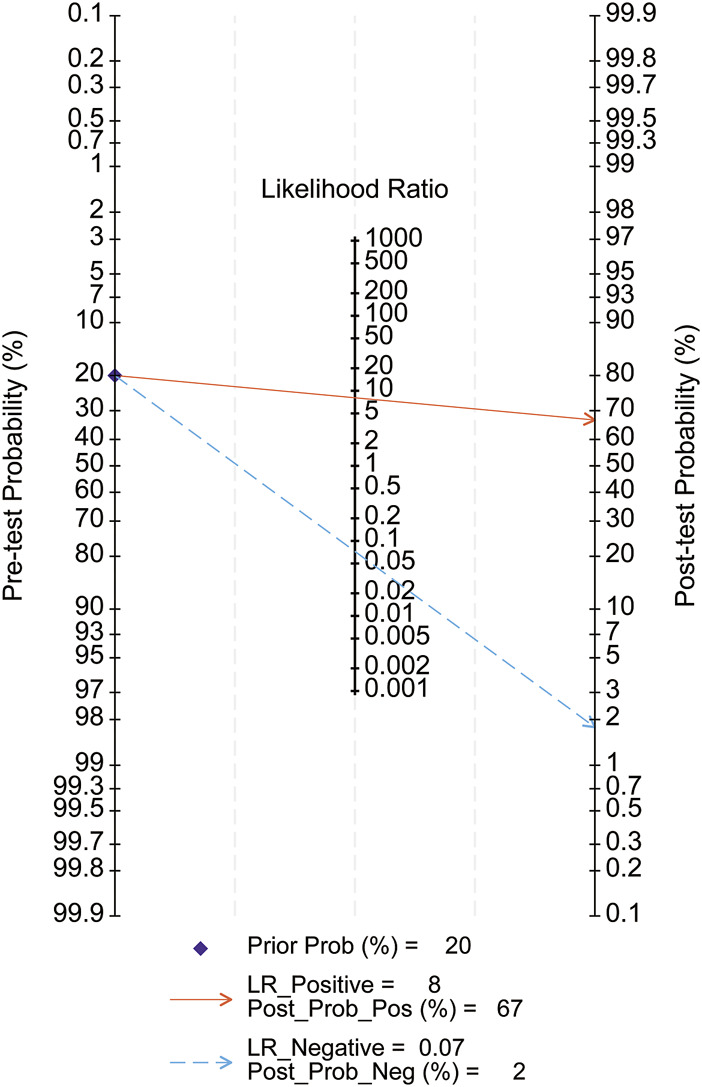
Fagan nomogram. Pre-test Probability: Pre-test Probability; Post-test Probability: Post-test Probability; Prior Prob: Pre-test Probability; LR_Positive: Positive Likelihood Ratio; LR_Negative: Negative Likelihood Ratio; Post-Post_Pos: Post-test probability for a positive test; Post-Post_Neg: Post-test probability for a negative test.

Diagnostic performance metrics provided further insights. The positive likelihood ratio (PLR) was 8, indicating an 8-fold higher probability of positive test results in affected individuals compared to controls. With a 20% pre-test probability, the post-test probability for a positive result increased to 67%. Conversely, the negative likelihood ratio (NLR) was 0.07, reducing the post-test probability for a negative result to 2%. These results support MMP-7’s diagnostic accuracy and clinical utility for BA (Figure 12).

## Discussion

This systematic review evaluated 17 studies (across 13 publications) examining serum MMP-7 as a diagnostic biomarker for BA in infants. Meta-analysis yielded pooled estimates of 93% sensitivity, 85% specificity, a positive likelihood ratio (PLR) of 7.68, a negative likelihood ratio (NLR) of 0.08, and an area under the ROC curve (AUC) of 0.96. These results underscore the high diagnostic utility of serum MMP-7 for BA.

Our analysis included a larger sample size than prior systematic evaluations, mitigating data limitations identified in earlier research. However, the pooled specificity was slightly lower than that reported in previous studies. Most prior studies determined serum MMP-7 cut-off values by maximizing ROC curve metrics, resulting in substantial inter-study variability (0.49–67.4 ng/mL). Sensitivity, specificity, and ROC values for these cut-offs were further validated by Jiang et al. (Jiang et al., 2024) and Pandurangi et al. (Pandurangi et al., 2024), underscoring serum MMP-7’s diagnostic reliability for BA. Notably, Jiang et al. (Jiang et al., 2024) derived an optimal cut-off value of 28.1 ng/mL for infants aged 0–30 days through a day-age stratified analysis, reporting sensitivity, specificity, and AUC values of 86.4% (95% CI: 75.0–94.0), 95.5% (95% CI: 77.2–99.9), and 0.947 (95% CI: 0.902–0.992), respectively.

To assess inter-study heterogeneity, we performed meta-regression analyses. The results showed that the publication year was a significant contributing factor to heterogeneity (p = 0.007). Examined covariates included country of origin, publication year, total sample size, BA infant sample size, and median subject age. Sensitivity analysis, involving sequential removal of individual studies, demonstrated consistent parameter overlap, reinforcing the robustness of our findings. Subgroup analysis suggested that the publication year had a significant impact on the study’s heterogeneity. Specifically, the heterogeneity mainly originated from studies published in 2023 and later years. Subgroup analysis by publication year suggested that heterogeneity may stem from methodological advancements (e.g., new ELISA kits) or regional variations in diagnostic protocols. Subgroup analysis by study type, however, identified no heterogeneity sources. Thus, it is hypothesized that the observed heterogeneity is attributable to variations in publication years. Nevertheless, the heterogeneity could also arise from non-covariate factors. These factors include disease severity, variability in experimental reagents, and technical discrepancies in experimental protocols. While a more homogeneous study population might reduce heterogeneity, it also presents a risk of introducing selection bias. Furthermore, the incomplete reporting of experimental design details in some studies intensifies the difficulty of interpreting heterogeneity.

Fagan nomograms (Figure 12) illustrated pre- and post-test probabilities, offering additional diagnostic insights. For example, a 20% pre-test probability of BA decreased to 2% with a negative MMP-7 result and increased to 68% with a positive result. These findings reinforce the high diagnostic accuracy of serum MMP-7 for BA.

Despite its strong diagnostic performance, heterogeneity across studies precludes definitive establishment of serum MMP-7 as a standalone BA biomarker. Considering the multifaceted aetiology of BA, serum MMP-7 may need to be integrated with differential diagnosis and other auxiliary tests. One investigation demonstrated the utility of combining age-adjusted MMP-7 ratios with imaging parameters like the Liver-Psoas Apparent Diffusion Coefficient Ratio (LTPAR) from diffusion-weighted MRI (DW-MRI). This study reported negative predictive values (NPVs) of 91.49% for LTPAR and 94.17% for age-adjusted MMP-7, underscoring the benefit of integrating serum and imaging data to enhance diagnostic accuracy in cholestatic infants (Wu et al., 2024). Additional studies have investigated MMP-7 combinations with biomarkers like IL-8 and GGT. AUC values for MMP-7 + IL-8, MMP-7 + GGT, and MMP-7 + IL-8+GGT were 0.8248, 0.9382, and 0.9392 (Wu et al., 2022), respectively—slightly lower than this meta-analysis’ AUC of 0.97. Although these results indicate that combined biomarkers have not yet matched MMP-7’s standalone diagnostic accuracy, further research is required to confirm their feasibility and reliability.

This systematic review has several limitations. First, most included studies lacked pre-specified cut-off values, had unbalanced sample sizes, and were predominantly conducted in China, limiting international generalizability. Second, substantial heterogeneity was observed across included studies. To address these limitations, future research should prioritize large-scale international studies and reagent standardization. Such efforts will facilitate further validation of serum MMP-7’s diagnostic performance for BA. Additionally, age-stratified patient analyses and subgroup evaluation of MMP-7’s diagnostic accuracy will help establish clinically relevant thresholds. Expanding combined diagnostic studies and exploring multimodal diagnostic models will also improve early BA detection accuracy.

In conclusion, serum matrix metalloproteinase-7 (MMP-7) demonstrates promise as a diagnostic biomarker for BA, with potential for inclusion in international diagnostic guidelines. However, future research must adhere to the Standards for Reporting Diagnostic Accuracy (STARD) guidelines (Bossuyt et al.) to design standardized, high-quality, large-sample multicenter studies. Such studies are essential to validate its diagnostic accuracy and define its clinical role.

## Data Availability

The original contributions presented in the study are included in the article/Supplementary Material, further inquiries can be directed to the corresponding author.

## Supplementary material

SUPPLEMENTARY TABLE S1 The characteristics of included studies. BA, biliary atresia; TP, true positive; FP, false positive; FN, false negative; TN, true negative; US, United States. UK, United Kingdom of Great Britain and Northern Ireland; RS, retrospective study; PS, prospective study, CSS, cross - sectional study.

## Abbreviations

BA, Biliary Atresia; MMP-7, Matrix Metalloproteinase 7; QUADAS-2, Quality Assessment of Diagnostic Accuracy Studies-2; AUC-ROC(AUC/ROC), Area under the receiver operating characteristic curve; PLR, Positive Likelihood Ratio; NLR, Negative Likelihood Ratio; DOR, Diagnosis Odds Ratio; TP, True Positive; FP, False Positive; FN, False Negative; TN, True Negative; SPENS, Sensitivity; SPEC, Specificity; LOGIT_SENS, log-odds transformation of sensitivity; LOGIT_SPEC, log-odds transformation of specificity; Elisa, Enzyme-Linked Immunosorbent Assay; LMA, Millipore Luminex Multiplex assay; TR-FRET, Time-Resolved Fluorescence Energy Transfer; ESS, Effective Sample Size; Post-Post_Pos, Post-test probability for a positive test; Post-Post_Neg, Post-test probability for a negative test; LTPAR, Liver-Psoas Apparent Diffusion Coefficient Ratio; DW-MRI, Diffusion-weighted magnetic resonance imaging.
