# Supplementary material for: Effect of serum MMP-7 on the diagnostic accuracy of biliary atresia: systematic review and meta-analysis
Source: Front Pharmacol. 2025 Jun 26;16:1581053. doi: 10.3389/fphar.2025.1581053 (PMC12240794; doi:10.3389/fphar.2025.1581053)
Supplement: Supplementary file 1 [file Table1.docx]

| **Author** | **Year** | **Country** | **Type** | **Cycle** | **Methods** | **Control** | **Total** | **BA** | **Gender（Male）** | **Median Age (BA, Days)** | **Cutoff (ng/mL)** | **TP** | **FP** | **FN** | **TN** |
| --- | --- | --- | --- | --- | --- | --- | --- | --- | --- | --- | --- | --- | --- | --- | --- |
| Chi | 2022 | China | PS | 2017-2020 | Elisa | Obstructive jaundice | 440 | 214 | 210 | 50 | 26.73 | 207 | 9 | 7 | 217 |
| Jiang | 2019 | China | PS | 2017-2018 | Elisa | Obstructive jaundice | 288 | 187 | 157 | 59 | 10.37 | 178 | 7 | 9 | 94 |
| Jiang | 2024 | China | RS | 2018-2019 | Elisa | Non-BA cholestasis | 318 | 172 | 187 | NA | 18 | 160 | 9 | 12 | 137 |
| Jiang | 2024 | China | PS | 2020-2022 | Elisa | Non-BA cholestasis | 687 | 395 | 399 | NA | 18 | 379 | 37 | 16 | 255 |
| Jiang | 2024 | China | PS | 2020-2022 | Elisa | Non-BA cholestasis | 81 | 59 | NA | NA | 28.1 | 51 | 1 | 8 | 21 |
| Lertudomphonwanit | 2017 | US | PS | NA | Elisa | Non-BA cholestasis | 70 | 35 | 35 | 62 | 0.49 | 34 | 3 | 1 | 32 |
| Rohani | 2022 | Iran | PS | 2018-2020 | Elisa | Non-BA cholestasis | 54 | 22 | 29 | 50 | 7.8 | 21 | 2 | 1 | 30 |
| Sakaguchi | 2022 | JP | RS | 2017-2020 | Elisa | Non-BA cholestasis | 47 | 27 | 21 | 56 | 18.6 | 27 | 2 | 0 | 18 |
| Singh | 2022 | India | PS | 2019-2020 | Elisa | Neonatal hepatitis | 46 | 25 | 34 | 87 | 4.99 | 24 | 2 | 1 | 19 |
| Wu | 2019 | China | RS | 2008-2018 | Elisa | Non-BA cholestasis | 100 | 36 | 62 | 42.36 | 1.43 | 35 | 11 | 1 | 53 |
| Yang | 2018 | China | PS | 2016-2018 | Elisa | Non-BA cholestasis | 135 | 75 | 62 | 54 | 52.85 | 74 | 3 | 1 | 57 |
| Karbasian | 2023 | Iran | CSS | 2020-2021 | Elisa | Hyperbilirubinemia | 44 | 13 | 23 | 69 | 1.8 | 11 | 17 | 2 | 14 |
| Aldeiri | 2023 | UK | RS | NA | Elisa | Non-BA cholestasis | 59 | 32 | NA | 56 | 69 | 22 | 2 | 10 | 25 |
| Xu | 2024 | China | RS | 2019-2022 | Elisa | Non-BA cholestasis | 68 | 44 | 25 | 58 | 3.138 | 27 | 3 | 17 | 21 |
| Pandurangi | 2024 | USA | PS | NA | LMA | Non-BA cholestasis | 399 | 201 | 221 | 64 | 52.8 | 189 | 44 | 12 | 154 |
| Pandurangi | 2024 | USA | PS | NA | LMA | Non-BA cholestasis | 399 | 201 | 221 | 64 | 67.4 | 182 | 31 | 19 | 167 |
| Pandurangi | 2024 | USA | PS | NA | TR-FRET | Non-BA cholestasis | 396 | 199 | NA | NA | 18.2 | 188 | 67 | 11 | 130 |
